# Necrosis of the Right Inferior Maxillary Bone

**Published:** 1864-05

**Authors:** J. Richardson

**Affiliations:** Terre Haute, Ind.


### NECROSIS OF THE RIGHT INFERIOR MAXILLARY BONE.

BY. J. RICHARDSON, D. D. S.

CASE. Active, intelligent, German boy, æt nine years; nervo-lymphatic temperament; strumous habit. During the month of August, 1862, was attacked with herpes circinatus, or ring-worm, affecting the right cheek. After the lapse of two or three months, during which time he had been subjected to medical treatment, the eruption suddenly disappeared on the use of some local application to the diseased surface, and simultaneously with its recession, the patient was seized with severe pain in the teeth of that side, while the gums and cheek became much swollen, the pain in the teeth continuing for some two weeks. At the end of this time, the temporary and first permanent molars became very loose from the disease which had so far invaded the maxillary bone as to indicate their removal, which was accordingly done.

The case came under my charge in November following. The portion of the jaw from which the teeth were taken was denuded and necrosed to something more than the depth of the sockets, but remained firmly adherent. Great tumefaction of the cheek, internally and externally, existed, with induration of the parts at the base and angle of the jaw, and limited capacity to open the mouth. The disease at this time was unaccompanied with pain; patient somewhat anemic, but general health fair and appetite good.

The physician in charge of the case had prescribed constitutional remedies comprising alteratives and tonics, and had used, in conjunction with these, some acid mixture as a solvent to the necrosed portion of bone. The latter treatment, as being not only useless but pernicious, was abandoned,—the former, with some modifications, continued. The parts affected were kept well cleansed with detergent washes, and occasional poultices and fomentations applied externally to the cheek. For the two or three succeeding months no material change occurred except a gradual extension of the disease towards the base of the jaw, and posteriorly into the angle of the same. Believing that a successful issue could only be hoped for by establishing a line of demarkation between the living and dead portions of bone, and exfoliation of the latter, and that this could be best effected by constitutional remedies and time, I had given no encouragement to the frequently expressed desires of the physician and parents to remove the dead portions by operation with instruments, believing that such a procedure could have no influence in either promoting or establishing a cure. The anxiety of the father of the boy, however, to have *something* done towards getting rid of the necrosed portion exposed to view, induced me finally to accede to his wishes, and neither his physician nor myself having bone forceps of suitable pattern for the operation, I gave him a letter to a distinguished surgeon of Cincinnati who operated for its removal before the medical class at St. John's Hospital, in the fore part of March, 1863. The father returned with the boy in a few days, elated and hopeful of results. All accessible portions of dead bone had been thoroughly removed, but it required no great amount of surgical acumen to divine that it yet remained for the efforts of the system, aided by judicious treatment, to accomplish what surgical skill was impotent to effect without absolute exsection of the jaw. The removal at this time of the family physician to a distant place, brought the patient exclusively under my control. As constitutional remedies, I prescribed, for the most part, syrup of iodide of iron in combination with the compound syrup of sarsaparilla; moderate exercise in the open air, and a generous diet. Topically, careful cleansing of the parts by injections of simple tepid water, dilute tinct. myrrh, and occasionally a solution of chlorinated soda. Careful examinations of the case were made from time to time, and it was soon evident that necrosed portions remained after the operation. In a month or six weeks thereafter, decided mobility of the remainder of the bone not operated upon was discovered, and seizing it with a pair of root forceps, I removed by careful traction, a thin layer of bone an inch or more in length, forming the base, and which comprised all that remained of the body of the jaw originally affected and subsequently operated upon. There was now no necrosed portion visible except a small piece fronting the angle of the jaw, but on lifting loose flaps of tumefied gum overhanging it, dead portions were discoverable extending some distance into the ramus, but firmly adherent to the living portions beyond. The constitutional treatment was continued and exfoliation waited for, examinations of its condition being frequently made.

About the middle of October last, I found evidence of complete detachment existing, and having made what dissections I could with the lancet from around the base of the dead bone, and having enlarged the opening in front somewhat, I seized the anterior exposed portion of the mass with root forceps, and by gentle but persistent manipulation succeeded in bringing it away entire. The portion removed embraced the entire angle of the jaw,—this, with the former portion removed, involved the complete loss of the maxillary bone from a point corresponding with the anterior bicuspid tooth to a point near the bifurcation formed by the coronoid and condoloid processes, or more than two-thirds of the right half of the jaw. In neither case was the removal of the fragments accompanied with severe pain or any considerable hemorrhage.

It is now some six months since the last portion was taken away. The tumefaction of the cheek has almost entirely disappeared; the parts within have closed up and present a perfectly healthy appearance; free action of the jaw; general health and appetite good. The external contour of the jaw is a little fuller and more rotund from the deposit of new osseous material at the base. The anterior bicuspid was erupted during the course of the disease, and now occupies its place in the arch, firm and apparently uninjured. The teeth posterior to the bicuspid are of course lost.

In connection with this case, an inquiry of considerable practical interest naturally suggests itself. What influence did the sudden suppression of the surface eruption have in the development of necrosis of the jaw? If they bore the relation of cause and effect, as they manifestly seem to have done, the case affords another illustration of the danger of working injury to other and more vital organs by a too active topical treatment of eruptions and other forms of surface disease.

*Terre Haute, Ind., April,* 1864.

